# The Implementation Process Assessment Tool: translation, contextualization, and psychometric evaluation of a Swedish version in a municipal elderly care context

**DOI:** 10.1186/s12913-024-11889-x

**Published:** 2024-11-13

**Authors:** Monica Kaltenbrunner, Heidi Hagerman, Cecilia Fagerström, Miriam Hartveit, Espen Nordheim, Mirjam Ekstedt

**Affiliations:** 1 Department of Health and Caring Sciences, Faculty of Health and Life Sciences, Linnaeus University, Universitetsplatsen 1, Kalmar, 39182 Sweden; 2 Faculty of Health and Occupational Studies, University of Gävle, Gävle, Sweden; 3 Department of Research, Region Kalmar County, Kalmar, Sweden; 4 Department of Research and Innovation, Helse Fonna Local Health Authority, Haugesund, Norway; 5 Department of Global Public Health and Primary Care, Faculty of Medicine, University of Bergen, Bergen, Norway; 6 Norwegian Centre for E-Health Research, University Hospital of North Norway, Tromsø, Norway; 7 Department of Learning, Informatics, Management and Ethics, Karolinska Institutet, Stockholm, Sweden

**Keywords:** CFIR, Elderly care, Factor analysis, Healthcare staff, Implementation, Instrument, IPAT, Validation, Questionnaire

## Abstract

**Background:**

The number of older adults with complex healthcare needs is growing alongside limited resources available in health services. To meet this challenge, it is urgent that healthcare staff are motivated and able to continuously translate new knowledge and working methods into daily practice. To facilitate such implementation, supportive measures responding to the healthcare personnel’s needs seem essential. The present study aims to translate, contextualize and test a Swedish version of the Implementation Process Assessment Tool (IPAT) for measuring the facilitation needs among staff implementing a new working process in municipal elderly care.

**Methods:**

A mixed-method design was used. First, the existing instrument was translated into Swedish. Thereafter, twelve staff members with different professions working in healthcare and at the municipal elderly care were interviewed using Think-aloud interviews to contextualize and test the face validity of the translated instrument. Lastly, the adjusted instrument (Swe-IPAT) was psychometrically evaluated through a cross-sectional survey among 305 staff members working in municipal elderly care.

**Results:**

The psychometric evaluation of the Swe-IPAT revealed satisfying properties. Three factors, largely in line with the original IPAT, are suggested. Internal consistency assessed using Cronbach’s alpha was 0.93 for the factor *individual phases for behavioral change and perception of the intervention*, 0.84 for the factor *individual activities*, and 0.95 for the factor *collective readiness and support*.

**Conclusions:**

The 27-item Swe-IPAT, translated into Swedish and contextualized, demonstrated satisfactory psychometric properties when tested in an elderly care context. The instrument is suggested to be useful in providing feedback to managers in tailoring support and assessing implementation efforts among healthcare staff in elderly care. However, more research is needed to evaluate its properties throughout the entire implementation process and to test the usability of Swe-IPAT in other settings.

**Supplementary Information:**

The online version contains supplementary material available at 10.1186/s12913-024-11889-x.

## Background

Most countries worldwide are experiencing growth in both the number of older adults and their proportion in the population. Aging leads to a gradual decline in physical and mental capacity and a growing risk of diseases [1, 2]. The rising number of people with complex healthcare needs, in combination with the decline in the working-age population, calls for rapid and efficient changes to healthcare services [3]. Efficient implementation of evidence-based practices, smart home devices, and coordinated care interventions are essential to ensure high-quality care for older adults in the future. To meet the upcoming challenges, it is necessary that organizations at different levels continuously improve, implement, integrate and sustain new, smarter, and more effective practices [4]. Although proven beneficial, implementation of innovations and new practices in standard care takes too long [5–10], and sustaining improved health processes is challenging [4, 11–13].

### Measuring the implementation process and system factors

Implementation of innovations or work processes is influenced by a large range of factors, including contextual, cultural, and economic aspects facilitating or hindering implementation. Theoretical frameworks enable enlightening the complex relationships between individual barriers and enablers involved in implementation, which might determine if an intervention works or not [14]. According to the Consolidated Framework for Implementation Research (CFIR) [8, 15], it is important to understand the care providers’ perceptions of intervention characteristics, the outer and inner setting support and facilitation, the characteristics of the individuals trying to perform the implementation, and the process of implementation. The care providers’ *readiness for change*, i.e., to what degree they are inclined and ready to undertake behavioral changes [16] and the *stages of change,* from awareness and insight to implementation and sustainment [17–19], are emphasized in particular. Barriers to implementation may be the intervention’s poor fit to the context, as well as system factors such as culture, poor communication, and lacking readiness for implementation among staff [20]. Quantifying care providers’ perceptions of these factors is important for two main reasons. First, empirical validation of the factors’ importance for implementation achievements contributes to the science of implementation. We need a better understanding of the factors’ individual and collective impacts during implementation processes, in various settings, and for the implementation of various practices or innovations. Second, to improve the speed and rate of implementation, we need information on how these factors are perceived in a given situation, to enable tailoring of supportive implementation activities and strategies to the implementers’ needs. Implementation efforts where intervention theories are employed to enable such tailoring of interventions are found to be more successful than others [21]. Data on the implementers’ perceptions can facilitate “just in time” and “just what we need” interventions [8, 22, 23].

Instruments have been developed to quantify the proposed facilitating or hindering implementation factors. Limitations of many of these instruments relate to unknown or limited psychometric properties [24–26], covering only the pre-phase of the implementation process or single theories [16], or being specific to one setting or intervention [27, 28]. Other instruments do not include the staff perspective [29] or are less feasible as questionnaires due to length, with an example being the 98-item instrument by Lehman et al. [30]. The Implementation Process Assessment Tool (IPAT) is a 27-item questionnaire for care providers to score how they perceive an ongoing implementation process. The IPAT is theoretically grounded in the CFIR framework, with particular emphasis on the stages of change [17–19] and individual and collective readiness for change theory [8]. Attributes in favor of the IPAT, in comparison with other instruments, are that it was developed in a healthcare setting, can assess changes over time, and is found to be positively associated with implementation outcomes [22, 31]. Another important aspect to consider when selecting an instrument is whether it was developed in a context similar to where it will be used [32]. This was the case with the IPAT. Studies have found the IPAT to be a useful instrument when assessing implementations [22, 33]. The IPAT has been used in a range of healthcare settings, as reported for instance in a protocol paper, focusing on general practitioners implementing a new guideline for shoulder pain [34], and a study on implementation of antibiotic stewardship programs in nursing homes (not yet published).

Although the investigations into the properties of the IPAT show promising results, its usefulness in implementing other practices in other settings and languages remains unknown. The theoretical grounding is expected to be relevant across healthcare services and countries. However, it was developed and tested in a specialized mental healthcare context in Norway, in the Norwegian language, among staff with at least a bachelor’s degree [31]. Contextual adaptation and investigation of the properties of the IPAT are essential when translating it for use in Swedish elderly care, and for implementation of other practices, among staff members less educated than in the Norwegian case. Thus, the aim of the present study is to translate, contextualize, and investigate the psychometric properties of a Swedish version of the IPAT when used during implementation of new working practices in municipal elderly care.

## Method

The translation and psychometric evaluation of the Norwegian IPAT to a Swedish context was performed in three steps: 1) Translation, 2) Contextualization, 3) Psychometric evaluation. For each step of this process, the sample and recruitment are described in detail.

An explorative design was chosen due to the instrument being tested in a new setting and practice and being translated into Swedish. The study was approved by the Swedish Ethical Review Authority (protocol code 2020 -01219, new application: 2022–02479-01).

### The IPAT

The IPAT, developed by Hartveit and colleagues [31], included four subscales: *individual stages for behavioral change* (items 1, 2, 3, 4, 5, 7, 20), *individual activities and perceived support* (items 8, 9, 10, 11, 14, 15, 18), *collective readiness and support* (items 6, 21, 22, 23, 24, 25, 26, 27), and *individual perceptions of the intervention* (items 12, 13, 16, 17, 19). All items, except one, have response alternatives ranging from 0 (not agree/not true) to 5 (agree/correct). Items 8–11 also have the response alternative “not relevant yet.” The one item (item 20) with other response alternative lists five statements as response options. Only one response alternative can be selected and the options range from early stage of change (1) to late stage of change (5) [31].

The IPAT was originally tested in a specialized mental healthcare setting and mainly among nurses, but also physicians, psychologists, social workers, and other professionals. When originally tested, the IPAT showed satisfactory psychometric properties. The internal consistency was assessed using Cronbach’s alpha, showing acceptable values ranging between 0.83 to 0.95 for the four subscales, and 0.96 for the total instrument. The risk of ceiling or floor effects was considered low, as there was a large variation in the scores [31]. A ceiling or floor effect occurs when scores cluster predominantly near the highest or lowest possible values, respectively. Such reduced score variability may occur because the response options fail to capture true differences, which can lead to less reliable results [35]. Permission to translate and test a Swedish version of the IPAT was obtained from the developer of IPAT.

### Step 1, translation

The original version of the instrument was first translated from Norwegian into Swedish [36] by HH, ME, CF, and ESN. A professional translator reviewed the translated instrument. Thereafter, discrepancies were discussed in the research group and with the translator, after which adjustments were made. Then, a back translation from Swedish to Norwegian was performed by a bilingual social worker, working in healthcare, who did not have access to the original version of the instrument. The research group discussed the back-translated version before further adjustments to the instrument were made [36].

### Step 2, contextualization

To contextualize and test the face validity of the translated instrument, cognitive interviews in the form of Think-aloud interviews (TAs) were conducted [36].

#### Sample and recruitment, Think-aloud interviews

The TAs were conducted in healthcare organizations in the south of Sweden. Managers in these organizations authorized the interviews. The inclusion criterion was that each participant had worked with a new practice/method/routine in the six months preceding their TA. The aim was to recruit a sample with variations regarding professions, workplaces, and experiences of implementations. The purposive sample, guided by the descriptions of Beatty and Willis [37], consisted of 12 participants in total. The first TAs were conducted in February–May 2021 with four registered nurses and one physician, working at three primary care centers, and two registered nurses working at a hospital. As the instrument needed to be adjusted to suit staff working at nursing homes in the municipality, further TAs were conducted in November 2021 with five licensed practical nurses (LPN) in elderly care.

#### Procedure, Think-aloud interviews

**Fig. 1:**
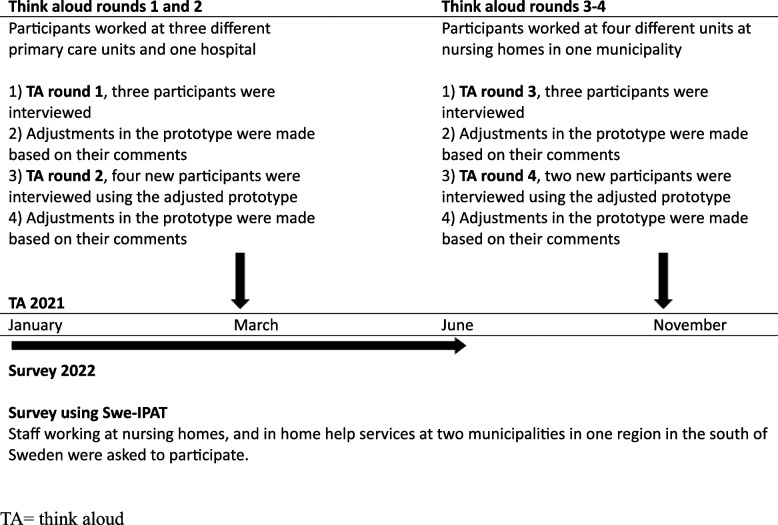
Flowchart of TAs conducted

The participants were asked to Think-aloud when reading the text and items in the prototype [38]. During the TAs, notes were taken on the participants’ interpretations, reactions, and comments on the text. When the participants showed signs that the text was difficult to read or interpret, the researcher asked questions such as “It seems that you are reading the text several times, what are your thoughts about the text?” [37]. Due to the Covid-19 pandemic, interviews were performed digitally. In total, four TA rounds were conducted, with new participants in each round. The instrument was adjusted after each TA round (Fig. 1). The TA rounds were terminated when no new information was revealed, as suggested by Collins [38]. As a last step, discrepancies between the adjusted instrument and the original IPAT were discussed in the research group and adjustments were made until consensus was reached.

### Step 3, psychometric evaluation

#### Sample, recruitment and procedure, survey

At this stage, the instrument was named the Swe-IPAT. A new sample was recruited to test the Swe-IPAT’s validity and reliability. Two municipalities in one region in the south of Sweden were asked to participate. In one municipality, the included staff worked at nursing homes, and in the other, the included staff worked in home help services. A convenience sample of staff members was recruited. To be included, a staff member should have worked at a unit and been affected by a new practice/method/routine in their daily work, for at least three months. The first-line managers informed all staff members about the study, both verbally and in writing. The Swe-IPAT was sent to eligible staff through their work e-mail. Data collection started in January 2022 and ended in June 2022. Two reminders were sent, and the last reminder included a paper version of Swe-IPAT.

### Data analysis

#### Think-aloud interviews

The data from the TAs were analyzed deductively and arranged into four categories, as suggested by Tourangeau [39]. The first category, *comprehension,* relates to whether words or phrases can be understood. The second, *retrieval,* concerns responding – for instance, whether or not information needed to respond is available. The third, *judgment,* involves whether it is difficult to put information together to respond. The last category, *response,* refers to whether the responder has difficulties selecting a response alternative [39].

#### Survey data

IBM SPSS Statistics version 27 was used to analyze survey data. Between 64 to 78 responses were replaced in each item. Thereafter, internal missing values were handled by removing responders with missing values of more than 10%. In total, the dataset dropped from 305 to 290 participants. For the remaining 290 participants, missing values were handled using multiple imputation (MI) [35]. Due to the reconceptualization of the IPAT, which encompassed several aspects beyond just language translation, we chose to conduct an exploratory factor analysis (EFA) to investigate potential underlying constructs in the Swe-IPAT. There is a lack of consensus in the literature regarding satisfactory sample sizes when conducting factor analysis [40–42]. Some studies suggest sample sizes as small as 50 participants, whereas others recommend up to 300 participants for factor analysis. Moreover, the recommended number of respondents per item varies widely, from 3 to 20 [40–42]. Given this variability, we considered our sample size satisfactory for testing the 27-item Swe-IPAT. The EFA provided us with a scree plot where the number of factors was determined based on eigenvalues. An eigenvalue greater than 1 was accepted [43]. Next, a principal axis factoring was performed. As the variables were considered to be correlated, promax rotation was selected. Bartlett's test of sphericity and Kaiser–Meyer–Olkin's measure of sampling adequacy (KMO) were used as goodness-of-fit indicators. The desired value in Bartlett's test is *p* < 0.05 and that for KMO is > 0.5 [44]. However, KMO > 0.8 is more typical. Items were sorted into the factor for which they had the highest loading. Lastly, internal validity was assessed using Cronbach’s alpha coefficient. A *p*-value < 0.05 was considered statistically significant. Floor and ceiling effects were assessed descriptively and by examining variations in score means and standard deviations (SDs).

## Results

### Sample characteristics and demographic data

Twelve individuals participated in TAs. The majority were women (*n* = 11) with a mean age of 43.58 years (SD = 10.20). The most common profession was registered nurse (*n* = 6). In the survey, the response rate was 61% (*n* = 305 of 502). Most participants were women, 87%, (*n* = 261), with a mean age of 43.80 years (SD = 12.27). The most common professions were LPN and nurse aide, 79% (*n* = 243) (see Table 1 for sample characteristics).

**Table 1:** Sample characteristics and demographic data of participants in the Think-aloud interviews and the web survey

	Think-aloud interviews	Survey
Total number of participants, n	12	305
Unit 1/unit 2		265 (87%)/40 (13%)
Women, n (%)	11	261 (87%)
Age (years)		
Mean (SD)	43.58 (10.20)	43.80 (12.27)
Md (Q_1_–Q_3_)	45 (32–52)	45 (35–54)
Profession, n		
Licensed practical nurse (LPN)	5	221 (72%)
Nurse aide	-	22 (7%)
Registered nurse	6	18 (6%)
Manager	-	12 (4%)
Physiotherapist	-	8 (3%)
Occupational therapist	-	10 (3%)
Social welfare officer	-	6 (2%)
Physician	1	-
Years worked at the present unit		
Mean (SD)	6.08 (8.40)	6.06 (7.04)
Md (Q_1_–Q_3_)	3 (2–7)	4 (2–7)
Years worked in the profession		
Mean (SD)	16.17 (10.62)	14.67 (11.25)
Md (Q_1_–Q_3_)	13 (9–26)	12 (5–23)
Permanent employee	12	271 (89%)/29 (10%)
Working full-time/part-time	9/3	198 (65%)/101 (33%)

*SD* Standard deviation, *Q*_*1*_*–Q*_*3*_ Quartiles 1–3. Where numbers do not add up to *n* = 305 or 100%, this is due to some participants not having responded to the questionnaire

### Translation and contextualization

During the TAs, the area most commented on by participants in the rounds was comprehension. Difficult words mentioned included “continuous,” “key person,” “expediency,” and “implementation.” Although all participants stated that the instrument was easy to understand and respond to, some said that it would benefit from being shorter and using simpler words. The instrument was adjusted after each TA round based on the participants’ comments and simpler words being chosen. No items were discarded. After the final adjustments, the instrument, now named the Swe-IPAT, underwent psychometric evaluation.

### Psychometric evaluation

In the survey, most of the participants (97%) responded to at least 13 of the 27 items in the Swe-IPAT. Internal missing values for each item were low, ranging from 1 to 5% (Table 2). Most missing values were seen for items 2, 18, 20, and 21 (5%). The lowest mean score was found for item 14, *I find I get necessary facilitation from management to succeed in the improvement work* (M = 3.21 (SD = 1.56)), and the highest for item 1, *I am aware that our unit will make efforts to improve (practice) support* (M = 4.23 (SD = 1.30)). For two items, regarding awareness (item 1) and willingness (item 6), more than half of the responders scored 5, potentially indicating a ceiling effect.

**Table 2:** Descriptive data for each item. The instrument is divided into four factors, as suggested in the original IPAT

	**Response alternative**									
*n*=305	0 = not agree/ not true	1	2	3	4	5 = agree/ correct	Not relevant yet	**Missing ** ***n* (%)**	**Mean (SD)**	**Md (Q**_**1**_–Q_**3**_**)**
**Factor 1**										
1) I am aware that our unit will make efforts to improve ___ support.	11 (3.6%)	8 (2.6%)	13 (4.3%)	25 (8.2%)	52 (17.0%)	186 (61.0%)		10 (3.3%)	4.23 (1.30)	5 (4–5)
2) I have recently learned of a new method/practice for __ support that has interested me.	17 (5.6%)	14 (4.6%)	34 (11.1%)	41 (13.4%)	71 (23.3%)	114 (37.4%)		14 (4.6%)	3.64 (1.50)	4 (3–5)
3) I have considered consequences of this new way of working for my own work.	16 (5.2%)	11 (3.6%)	26 (8.5%)	45 (14.8%)	80 (26.2%)	116 (38%)		11 (3.6%)	3.73 (1.43)	4 (3–5)
4) I have discussed with colleagues how this new practice will work in our unit.	22 (7.2%)	18 (5.9%)	33 (10.8%)	47 (15.4%)	69 (22.6%)	106 (34.8%)		10 (3.3%)	3.49 (1.57)	4 (3–5)
5) I have considered the pros and cons of the new practice and believe the benefits will outweigh the effort.	22 (7.2%)	15 (4.9%)	34 (11.1%)	50 (16.4%)	74 (24.3%)	100 (32.8%)		10 (3.3%)	3.49 (1.54)	4 (3–5)
7) I make it clear to my colleagues that I want to work to improve __ support.	11 (3.6%)	11 (3.6%)	23 (7.5%)	37 (12.1%)	72 (23.6%)	141 (46.2%)		10 (3.3%)	3.94 (1.36)	4 (3–5)
20) Which of the following sentences describes you best in relation to __support efforts (…).		20 (6.6%)	64 (21.0%)	55 (18.0%)	33 (10.8%)	117 (38.4%)		16 (5.2%)	3.56 (1.39)	4 (2–5)
**Factor 2**										
8) I have changed my way of working to make my contribution to the new practice in __ support.	15 (4.9%)	12 (3.9%)	17 (5.6%)	24 (7.9%)	64 (21.0%)	91 (29.8%)	78 (25.6%)	4 (1.3%)	3.72 (1.52)	4 (3–5)
9) I provide constructive feedback to help us achieve the change.	9 (3.0%)	12 (3.9%)	20 (6.6%)	29 (9.5%)	59 (19.3%)	102 (33.4%)	70 (23.0%)	4 (1.3%)	3.83 (1.41)	4 (3–5)
10) I keep track of data we get at our unit’s performance to see how things are developing.	11 (3.6%)	13 (4.3%)	12 (3.9%)	26 (8.5%)	59 (19.3%)	116 (38.0%)	64 (21.0%)	4 (1.3%)	3.93 (1.43)	4 (3–5)
11) I remind myself and my colleagues about our new practice if we deviate from it.	13 (4.3%)	19 (6.2%)	18 (5.9%)	35 (11.5%)	67 (22.0%)	82 (26.9%)	67 (22.0%)	4 (1.3%)	3.58 (1.50)	4 (3–5)
14) I find that I get necessary facilitation from management to succeed in the improvement work.	26 (8.5%)	19 (6.2%)	43 (14.1%)	58 (19.0%)	72(23.6%)	74 (24.3%)		13 (4.3%)	3.21 (1.56)	4 (2–5)
15) I find that I get the necessary support from key colleagues to succeed in the improvement effort.	18 (5.9%)	25 (8.2%)	40 (13.1%)	50 (16.4%)	72 (23.6%)	87 (28.5%)		13 (4.3%)	3.35 (1.54)	4 (2–5)
18) I feel I am getting adequate support to enable me to carry out my part of the improvement.	20 (6.6%)	19 (6.2%)	39 (12.8%)	53 (17.4%)	86 (28.2%)	74 (24.3%)		14 (4.6%)	3.33 (1.49)	4 (2–5)
**Factor 3**										
6) I am willing to take on the necessary additional work to improve __ support.	9 (3.0%)	3 (1.0%)	14 (4.6%)	38 (12.5%)	65 (21.3%)	166 (54.4%)		10 (3.3%)	4.19 (1.20)	5 (4–5)
21) We who work here agree that we have potential for improvement in __ support.	11 (3.6%)	17 (5.6%)	38 (12.5%)	55 (18.0%)	86 (28.2%)	83 (27.2%)		15 (4.9%)	3.51 (1.38)	4 (3–5)
22) We agree that the proposed interventions are appropriate for realizing the improvement potential	12 (3.9%)	14 (4.6%)	31 (10.2%)	52 (17.0%)	91 (29.8%)	92 (30.2%)		13 (4.3%)	3.62 (1.37)	4 (3–5)
23) We all feel good about the improvement efforts in __ support.	15 (4.9%)	17 (5.6%)	39 (12.8%)	54 (17.7%)	83 (27.2%)	84 (27.5%)		13 (4.3%)	3.46 (1.45)	4 (3–5)
24) We have agreed to make every effort to implement __ support.	16 (5.2%)	16 (5.2%)	39 (12.8%)	47 (15.4%)	75 (24.6%)	99 (32.5%)		13 (4.3%)	3.53 (1.48)	4 (3–5)
25) We feel confident that we have the necessary knowledge and experience of systematic improvement work to bring about the desired change.	14 (4.6%)	20 (6.6%)	43 (14.1%)	55 (18.0%)	87 (28.5%)	74 (24.3%)		12 (3.9%)	3.38 (1.42)	4 (2–4)
26) We feel confident that our organization will involve everyone in this improvement work in __ support.	12 (3.9%)	17 (5.6%)	35 (11.5%)	49 (16.1%)	72 (23.6%)	107 (35.1%)		13 (4.3%)	3.62 (1.43)	4 (3–5)
27) In our view, management is committed to implementing and following up the results of the improvement work in __ support.	14 (4.6%)	10 (3.3%)	30 (9.8%)	62 (20.3%)	88 (28.9%)	88 (28.9%)		13 (4.3%)	3.59 (1.36)	4 (3–5)
**Factor 4**										
12) I believe we have a clear potential for improvement in our __ support.	7 (2.3%)	10 (3.3%)	41 (13.4%)	51 (16.7%)	77 (25.2%)	106 (34.8%)		13 (4.3%)	3.70 (1.31)	4 (3–5)
13) I believe that the efforts and the interventions are appropriate to improve our ___ practice.	10 (3.3%)	2 (0.7%)	34 (11.1%)	49 (16.1%)	75 (24.6%)	122 (40.0%)		13 (4.3%)	3.87 (1.28)	4 (3–5)
16) I believe the patients will benefit from the improvement.	8 (2.6%)	9 (3.0%)	24 (7.9%)	44 (14.4%)	74 (24.3%)	135 (44.3%)		11 (3.6%)	3.95 (1.28)	4 (3–5)
17) I believe the improvement will benefit me personally (e.g., saving time, increasing my confidence and enhancing predictability).	15 (4.9%)	15 (4.9%)	35 (11.5%)	55 (18.0%)	82 (26.9%)	91 (29.8%)		12 (3.9%)	3.52 (1.44)	4 (3–5)
19) I believe I will manage the effort and be able to comply with the new practice.	5 (1.6%)	6 (2.0%)	16 (5.2%)	50 (16.4%)	79 (25.9%)	137 (44.9%)		12 (3.9%)	4.04 (1.15)	4 (3–5)

The response alternative “not relevant yet” was merged with the response option 0 (not agree/not true) in the analysis

All items originate from the original IPAT instrument (Hartveit et al. 2019)

SD Standard deviation, Md Median, Q_1_–Q_3_ Quartiles 1–3

The EFA suggested three factors for the Swe-IPAT, largely in line with the four factors in the original IPAT (Table 3). Items were sorted into the factor for which they had the highest loading. Factor 1 in Swe-IPAT includes items 1–7, 12, 13, 16, 17, 19, and 20 and was labeled “individual phases for behavioral change and perception of the intervention.” The second factor includes items 8–11 and we labelled it “individual activities.” Factor 3 in Swe-IPAT includes items 14, 15, 18, and 21–27 and was labeled “collective readiness and support.”

**Table 3:** The final 3-factor structure and internal consistency in the Swe-IPAT and the original IPAT

	Factor loading Swe-IPAT Factor			Factor in original IPAT
α for total scale 0.96	1	2	3	
**Factor 1, **Individual phases for behavioral change and perception of the intervention *α = 0.93*				
Item 1	0.711		0.575	1
Item 2	0.788		0.514	1
Item 3	0.696		0.419	1
Item 4	0.736	0.554	0.443	1
Item 5	0.809	0.454	0.528	1
Item 6	0.751		0.482	3
Item 7	0.768	0.506	0.605	1
Item 12	0.719		0.527	4
Item 13	0.814		0.664	4
Item 16	0.800		0.674	4
Item 17	0.724		0.698	4
Item 19	0.694		0.559	4
Item 20	0.534	0.505		1
**Factor 2, **Individual activities *α = 0.84*				
Item 8		0.768		2
Item 9		0.877		2
Item 10		0.758	0.437	2
Item 11		0.828		2
**Factor 3, **Collective readiness and support *α = 0.95*				
Item 14	0.563		0.789	2
Item 15	0.573		0.866	2
Item 18	0.577		0.843	2
Item 21	0.579		0.807	3
Item 22	0.620	0.449	0.850	3
Item 23	0.622	0.402	0.851	3
Item 24	0.607		0.869	3
Item 25	0.546		0.828	3
Item 26	0.558		0.835	3
Item 27	0.602		0.846	3

α =Cronbach’s alpha

The results of the principal axis factoring with promax rotation justified the use of factor analysis and showed an acceptable model fit, as the KMO value was 0.941 and the value from Bartlett’s Test of Sphericity was significant (χ^2^ = 6186.953, df = 351, *p*-value < 0.001). Three factors had an eigenvalue > 1.0 and explained 64.1% of the variance in the data. Table 3 shows the factor loadings to each factor. Item 17 and Item 20 loaded almost equally to two factors (factors 1 and 3 and factors 1 and 2, respectively) and were placed in the factor with the highest loading, which was factor 1 in both cases. The internal consistency of the Swe-IPAT was satisfactory: Cronbach’s alpha values ranged from 0.84 to 0.95 for the three factors and was 0.96 for the total instrument (Table 3).

## Discussion

The present study aimed to translate, contextualize, and test the psychometric properties of a Swedish version of the 27-item IPAT, the Swe-IPAT. The Swe-IPAT was found relevant and easy to understand by representatives of staff in municipal elderly care, and psychometric properties showed promising results. The three sub-scales identified represented constructs known from existing implementation literature and were largely in line with the four sub-scales in the original IPAT [31]. The Swe-IPAT showed that staff members in Swedish elderly care reported a high degree of awareness, motivation, and self-confidence, and perceived low levels of facilitation and support. In general, most staff members were well-informed about the implementation of new work practices. The staff members were prepared to put in an effort to make the new practices work, saw their pros, and believed they could comply with them. However, some suggestions for improvements were made, for instance concerning perceived support from managers.

Evaluating ongoing implementations is valuable, especially as implementation strategies are generally adapted to the context and thus change over time [45]. For successful implementation of new work practices, we consider it imperative to gather feedback from staff on relevant aspects, to enable continuous modification of the implementation plan and support. Mental healthcare providers’ perceptions of the factors represented in the original IPAT have been found to correlate with implementation success, especially more than one year after the start of an implementation [22]. In the initial steps of the present study, the staff from municipality care services found the factors in the Swe-IPAT to be highly relevant for their setting. Other studies from a range of care contexts suggest that sufficient planning to engage and involve staff may distinguish high implementation success from low implementation success [46–49]. Involving staff in the planning of implementation, to ensure tailored facilitation and support, may result in higher commitment and preparedness to make changes and share the workload [46]. However, implementation strategies are often chosen without adequately considering the diverse settings that may encompass unique factors affecting the implementation process [49]. Feedback from staff on the perception of facilitating and hindering factors operationalized in the IPAT can be useful both for planning and conducting the implementation of a new procedure and for evaluating implementation success [14, 50].

### The three sub-constructs of the Swe-IPAT

The explorative factor analysis suggested three factors in the Swe-IPAT: “individual phases for behavioral change and perception of the intervention,” “individual activities,” and “collective readiness and support.” The original IPAT suggests four factors. However, cross-loading has been reported for the IPAT [31], i.e., several items were found to be associated with more than one factor. This was explained as being related to the complexity and interaction between factors, an aspect that is relevant also in the present study. According to Hartveit et al. [31], the impact of each factor when isolated is of less relevance for understanding implementation. Hartveit et al. [22] also concluded this in their study investigating the relationship between IPAT score and fidelity.

As mentioned, the present study suggests three factors, all representing acknowledged implementation constructs. Factor 1 “Individual phases for behavioral change and perception of the intervention” in the Swe-IPAT is a combination of factors 1 and 4 and item 6 from the original IPAT. The combination is not surprising, as the individual phases for behavioral change items are grounded in the Stages of Change theory [17–19], which emphasizes the cognitive assessment of pros and cons in deciding if one should contribute to the implementation. Factor 2, “Individual activities,” included items 8–11, meaning that items 14, 15, and 18 from the original IPAT structure were not included in this factor. The discarded items concern perceived support and facilitation loaded higher to factor 3 in the Swe-IPAT. Factor 3, “collective readiness and support,” included items 21–27, which also in the original IPAT were included in the factor representing collective readiness, as well as items 14, 15, and 18. Items 14, 15, and 18 all concern perceived support to proceed with an implementation effort. Such support, or the lack thereof, is a result of social interactions [8]. The shaping of collective perceptions is established by the same interactions. We understand factor 3 in the Swe-IPAT as the social interactions promoting a sense of contextual support and collective engagement and understanding, in line with the “Supportive climate” described by Holt et al. [51] as an element of organizational readiness.

### Strengths and limitations

There is a plethora of different instruments assessing implementation processes. However, some of these instruments have limitations due to fragmented theoretical grounding and/or psychometric properties [24–26]. One strength of the study is that the Swe-IPAT is based on a robust instrument constructed using well-defined implementation theories [31]. The involvement of the principal investigator from the original IPAT study in the instrument development for the present study facilitated the valid operationalization of the theoretical constructs in the new setting and language. The sample size was satisfactory and can be seen as a strength, as it increases the likelihood of accurate estimates [40–42]. The diversity in genders, ages, professions, and healthcare settings in the sample is positive for the generalizability of our findings and conclusions [35]. However, including frontline leaders in the sample, as we did, can be a limitation. Their opinions may be biased by their role in engaging subordinates and they are often more informed than others, which could imply a generally higher IPAT score [35].

Contextualization and face validity were tested using Think-aloud interviews. The number of participants in the TAs (*n* = 12) is in accordance with the suggestions for an acceptable sample size by Beatty and Willis [37]. Having this sample size increases the ability to capture different individuals’ perspectives on an instrument. The variation achieved in the TA sample meant that different professions in elderly care and healthcare interpreted the questionnaire. Knowing when to end TA rounds is a challenge. We ended after four TA rounds, when no new information was obtained [37]. According to the TAs, the Swe-IPAT was relevant and easy to interpret and respond to. This was corroborated by there being only a small amount of missing data.

This study also has some limitations. An aspect that needs to be highlighted is the generally high scores in the responses, although all response options were used. One reason for this can be that participants with a positive attitude to the intervention generally chose to participate and/or responded as they perceived to be desirable [35]. Another limitation is that we did not study test–retest reliability. However, this may not be applicable, as implementation is a dynamic process during which individuals may change their mindsets, which can give a false test–retest result. An assessment of differences between responders and non-responders was not possible in the study, as no information about non-responders was available. We have not tested the associations between Swe-IPAT scores and implementation outcomes. However, in this study, few changes in wording were made based on the TAs and the results from the EFA showed strong support for the three factors in the Swe-IPAT. This indicates that scores for the Swedish version may have similar associations as the original in relation to implementation outcomes (such as fidelity or compliance with the new practice). The generalizability of the Swe-IPAT to other Swedish healthcare settings and implementation efforts is unknown.

## Conclusion, clinical implications, and future research

We have translated, contextualized, and tested the psychometric properties of the 27-item Swe-IPAT in a municipal elderly care context. The Swe-IPAT shows acceptable psychometric properties. The instrument is suggested to be useful when assessing implementation processes among staff in elderly care in Sweden. The Swe-IPAT has the potential to deliver constructive feedback to managers and implementation teams regarding their endeavors to foster engagement among staff early in the implementation process. If used repeatedly throughout the process, the Swe-IPAT instrument could serve as a valuable tool to identify key focus areas that may be challenging in future phases of the implementation process or facilitate the implementation in general. Thus, it could be possible to tailor implementation support based on the specific needs of those participating in the implementation. However, further evaluation is needed.

We suggest future research focusing on testing the usability of the Swe-IPAT in other similar settings and studying its associations with implementation outcomes. We also suggest that future research focuses on leadership styles and staff-perceived support during the implementation process, as well as on the differences across stages of change within the organization.

## Supplementary Information

Supplementary Material 1.

## Data Availability

The interview data that were collected and analyzed in this manuscript are not publicly available due to participants not having consented to public availability. However, all the datasets and aggregated data in Swedish are available from the corresponding author on reasonable request. We have attached the Swe-IPAT as a supplementary file, and it is free for everyone to use. Please contact the team of authors via the corresponding author before using Swe-IPAT.
