# Supplementary material for: The Implementation Process Assessment Tool: translation, contextualization, and psychometric evaluation of a Swedish version in a municipal elderly care context
Source: BMC Health Serv Res. 2024 Nov 13;24:1391. doi: 10.1186/s12913-024-11889-x (PMC11558869; doi:10.1186/s12913-024-11889-x)
Supplement: Supplementary file 1 — Supplementary Material 1. [file 12913_2024_11889_MOESM1_ESM.docx]

**Swe-IPAT (the Swedish version of the Implementation Process Assessment Tool)**

Enkäten nedan är gratis att använda. Men vi ser gärna att ni kontaktar förste-författarna för att informera om att ni kommer att använda enkäten.

| 1. Jag känner till att vår enhet kommer att satsa på att förbättra för (vilka? personal, brukare, patienter) i och med (namn på det nya arbetssättet). | | | | | | |
| --- | --- | --- | --- | --- | --- | --- |
| 1. Jag har fått reda på ett nytt arbetssätt, (namn på det nya arbetssättet), som på senare tid väckt mitt intresse. | | | | | | |
| 1. Jag har funderat över vad det nya arbetssättet kommer att betyda för mitt eget arbete. | | | | | | |
| 1. Jag har pratat med mina arbetskamrater om hur det nya arbetssättet kommer att fungera hos oss. | | | | | | |
| 1. Jag har övervägt fördelar och nackdelar med det nya arbetssättet och tror att fördelarna väger upp arbetet som krävs. | | | | | | |
| 1. Jag är inställd på att göra det extra arbete som krävs för att förbättra för (vilka? personal, brukare, patienter) genom (namn på det nya arbetssättet). | | | | | | |
| 1. Jag är tydlig mot mina arbetskamrater att jag vill arbeta för att förbättra för (vilka? personal, brukare, patienter) genom (namn på det nya arbetssättet). | | | | | | |
| 1. Jag har ändrat hur jag arbetar för att göra min del i införandet av (namn på det nya arbetssättet). * | | | | | | |
| 1. Jag delar med mig av konstruktiv feedback för att vi ska kunna genomföra förändringen (skriv inom parentes namnet på det nya arbetssättet). * | | | | | | |
| 1. Jag håller mig uppdaterad om den rapportering vi får om enhetens resultat för att följa utvecklingen. * | | | | | | |
| 1. Jag påminner mig själv och mina arbetskamrater om vi avviker från vårt nya arbetssätt. * | | | | | | |
| 1. Jag anser att vi har en klar förbättringspotential när det gäller (namn på det nya arbetssättet). | | | | | | |
| 1. Jag anser att insatserna med att förbättra för (vilka? personal, brukare, patienter) genom (namn på det nya arbetssättet) är lämpliga. | | | | | | |
| 1. Jag upplever att ledningen ger mig de förutsättningar jag behöver för att kunna lyckas med förbättringsarbetet. | | | | | | |
| 1. Jag upplever att jag får det stöd jag behöver från viktiga nyckelpersoner för att kunna lyckas med förbättringsarbetet (uppge vilka nyckelpersonerna kan vara). | | | | | | |
| 1. Jag tror att (vilka? personal, brukare, patienter) kommer att få nytta av förbättringarna. | | | | | | |
| 1. Jag tror att förbättringen kommer att gynna mig (t.ex. spara tid, ge större trygghet eller bättre planeringsmöjligheter). | | | | | | |
| 1. Jag får tillräckligt stöd för att kunna genomföra min del av förbättringsarbetet. | | | | | | |
| 1. Jag tror jag klarar av insatsen som krävs för att arbeta enligt det nya arbetssättet. | | | | | | |
| 1. Vilken av dessa beskrivningar tycker du passar bäst in på dig när det gäller arbetsinsatsen för (namn på det nya arbetssättet). Välj endast ett påstående. | | | | | | |
|  | Jag har inte satt mig in i vad det här förbättringsarbetet handlar om. | | | | | |
|  | Jag har fått information om det nya arbetssättet och tycker det verkar intressant, men jag har inte tänkt mer på det än så. | | | | | |
|  | Jag är intresserad av förbättringar inom det aktuella området och tycker att vår enhet bör ändra arbessätt utifrån tagna beslut. | | | | | |
|  | Jag tycker att vår enhet bör ändra arbetssätt utifrån tagna beslut och förstår att detta kommer att innebära merarbete. Men jag anser att det är värt det. | | | | | |
|  | Jag deltar aktivt i att förändra arbetssättet genom att följa beslut, och jag vill medverka till att lösa problem i samband med förändringarna. | | | | | |
|  | | | | | | |
| Nu ber vi dig utvärdera förbättringsarbetet vid enheten där du arbetar. I vilken grad stämmer följande påstående? (Vi/vår enhet=vi som arbetar på den/det aktuella (t.ex. avdelningen, enheten, vård- och omsorgsboendet) | | | | | | |
| 1. Vi som arbetar här delar uppfattningen att vi har en förbättringspotential när det gäller (namn på det nya arbetssättet). | | | | | | |
| 1. Vi är överens om att det nya arbetssättet är lämpligt för att skapa förutsättningar för att förbättra för (vilka? personal, brukare, patienter). | | | | | | |
| 1. Vi känner tillsammans det positivt att få arbeta med (namn på det nya arbetssättet). | | | | | | |
| 1. Vi är överens om att satsa allt för att lyckas införa (namn på det nya arbetssättet). | | | | | | |
| 1. Vi känner oss trygga med att vi har nödvändig kunskap och erfarenhet av systematiskt förbättringsarbete för att uppnå den önskade förändringen (systematiskt förbättringsarbete kan vara att man på ett planerat sätt arbetar för att minska risken att göra fel i arbetet). | | | | | | |
| 1. Vi litar på att arbetsgivaren kommer att involvera alla i det förbättringsarbete som genomförs i och med (namn på det nya arbetssättet). | | | | | | |
| 1. Vi uppfattar att ledningen har åtagit sig att genomföra förbättringar i och med (namn på det nya arbetssättet) och se till att resultaten blir bestående. | | | | | | |
| Svarsalternativ: | | | | | | |
| Stämmer inte (0) | |  |  |  |  | Stämmer helt (5) |

* Fråga 8, 9, 10 och 11 har samma svarsalternativ som övriga frågor (förutom påståendefrågan) samt även svarsalternativet *ej aktuellt ännu*.
